# Characterization and Functional Analysis of Small Heat Shock Protein Genes (*Hsp22.2* and *Hsp26.7*) in *Sitodiplosis mosellana* Diapause

**DOI:** 10.3390/insects16070649

**Published:** 2025-06-20

**Authors:** Qitong Huang, Qian Ma, Xiaobin Liu, Keyan Zhu-Salzman, Weining Cheng

**Affiliations:** 1 Shandong Institute of Sericulture, Shandong Academy of Agricultural Sciences, Yantai 265503, China; 13760637249hqt@nwafu.edu.cn (Q.H.); bin0331@126.com (X.L.); 2 Key Laboratory of Plant Protection Resources and Pest Management of Ministry of Education, College of Plant Protection, Northwest A&F University, Yangling 712100, China; maqian981017@163.com; 3 Department of Entomology, Texas A&M University, College Station, TX 77843, USA

**Keywords:** small heat shock protein, diapause, *Sitodiplosis mosellana*, temperature stress, cold adaptation

## Abstract

Small heat shock proteins (sHsps) play crucial roles in organismal adaptation to stress tolerance. *Sitodiplosis mosellana*, a devastating insect wheat pest, undergoes long obligatory larval diapause to survive temperature extremes during summer and winter. To elucidate the function of sHsps in this process, two sHsp-encoding genes (*SmHsp22.2* and *SmHsp26.7*) were characterized from *S. mosellana*, and their responsiveness to diapause and thermal stress, as well as their roles in cold stress, was analyzed. Both *SmHsp22.2* and *SmHsp26.7* possessed the canonical α-crystallin domain and lacked introns. Quantitative PCR indicated significant upregulation of *SmHsp22.2* and *SmHsp26.7* during diapause, especially in summer and winter. Notably, *SmHsp22.2* exhibited higher expression in summer relative to winter, whereas *SmHsp26.7* showed the opposite profile. Moreover, short-term heat shock (≥35 °C) in over-summering larvae or cold shock (≤−10 °C) in over-wintering larvae was found to trigger transcriptional upregulation of both genes, while prolonged temperature extremes (i.e., 45–50 °C or −15 °C) did not elicit a comparable response. RNA interference-mediated knockdown of both genes significantly increased the mortality of *S. mosellana* larvae under cold stress. These findings indicate the importance of both *SmHsps* in diapause and environmental adaptation in *S. mosellana*.

## Simple Summary

Small heat shock proteins (sHsps) are crucial players not only in heat/cold adaptation in insects, but also in insect diapause. This study aimed to characterize two sHsp genes (*SmHsp22.2* and *SmHsp26.7*) from *S. mosellana*, a serious wheat pest that undergoes long obligatory larval diapause to survive temperature extremes during summer and winter, and determine their expression in association with diapause and thermal stress, as well as their roles in cold stress. The results showed that *SmHsp22.2* and *SmHsp26.7* were significantly upregulated during diapause including short-term heat/cold stress. RNAi-mediated knockdown of both genes significantly increased the mortality of *S. mosellana* larvae under cold stress. These results suggest that *SmHsp22.2* and *SmHsp26.7* form an integral part of adaptations to diapause in *S. mosellana*, and that their increased expression is essential for protecting *S. mosellana* from the adverse effects of low temperature.

## 1. Introduction

Diapause is a developmental tactic that ensures the survival of insects in extreme environments, synchronizing their life cycles with times of the year conducive to growth and reproduction [1,2]. Diapause can occur at various life stages according to the insect species [3,4,5,6], and involves significant physiological and metabolic shifts, including reductions in metabolic rates, augmentation of energy reserves, and enhanced resistance to stress [7,8]. Given its critical importance in insect growth and reproduction, unraveling the molecular underpinnings of diapause is paramount for formulating effective agricultural pest-control strategies [9].

Heat shock proteins (Hsps) are upregulated in response to various biotic and abiotic stresses, including pathogen invasion, heat, and cold [10,11,12,13]. Hsps function as molecular chaperones, and promote cellular protein homeostasis by binding and refolding stress-compromised proteins [14,15,16]. They are classified into various families according to evolutionary relationships and size, including the Hsp100, Hsp90, Hsp70, Hsp60, Hsp40, and small Hsp (sHsp) families [17,18,19].

sHsps are a large and ancient family of ATP-independent molecular chaperones with monomeric molecular weights between 12 and 43 kDa. Despite variations in their sequences and oligomer sizes, all sHsps contain a highly conserved core sequence of approximately 80–100 amino acids, the α-crystallin domain, which is bordered by non-conserved N- and C-terminal extensions [20,21]. The α-crystallin domain comprises a β-sandwich consisting of 7 to 8 antiparallel β-strands, and is responsible for the chaperone activity of sHsps [22]. Beyond their roles as chaperones, sHsps engage in a diverse array of biological processes, such as maintenance of the cytoskeleton [23], DNA repair [24], cellular apoptosis and autophagy [25], and membrane stability [26].

Recent studies have demonstrated the roles of sHsps in the regulation of insect diapause; however, their expression patterns differ markedly according to the insect species and sHsp family [27,28]. For example, in the blowfly *Lucilia sericata*, *Hsp23* transcript levels are not altered by diapause [29], in contrast to changes in *Hsp23* expression seen during pupal diapause in the flesh fly *Sarcophaga crassipalpis* [30] and larval diapause of the blowfly *Calliphora vicina* [31]. Furthermore, elevated expression of *sHsps* during diapause has been reported in a variety of insect species, including *Calanus finmarchicus Hsp22* [32], *Leguminivora glycinivorella Hsp19.8* and *18.9* [33], *Pieris melete Hsp19.5* and *20.0* [27], and *Ostrinia nubilalis Hsp20.1* [34]. In contrast, downregulation of *Sesamia nonagrioides Hsp20.8* and *Trogoderma granarium Hsp20.5* and *21.2* was observed during diapause [28,35]. These variations in expression suggest differences in sHsp function among insect species during diapause.

The orange wheat blossom midge *Sitodiplosis mosellana* (Géhin) (Diptera: Cecidomyiidae), a notorious pest of wheat across the northern hemisphere, causes substantial reductions in yield and economic damage during peak infestation years [36,37,38]. As a univoltine species, the mature larvae in northern China undergo obligatory diapause within cocoons in the soil during the 10 months from June to early April of the following year. The initiation of post-diapause development is triggered by the onset of increased ambient temperatures in the spring [39]. This prolonged diapause not only enables the survival of the insect during hot summer and cold winter temperatures but also ensures synchronization of its development with wheat growth cycles. While we have previously elucidated the expression patterns of *Hsp17.4* and *Hsp20.3* in *S. mosellana* diapause [40], the roles of other sHsps in regulating this process are still unclear.

In this study, we cloned and characterized two novel sHsp-encoding genes from the pre-diapause larvae of *S. mosellana*, and analyzed their expression profiles under conditions of diapause and thermal stress. In addition, the effects of *SmHsp22.2*/*Hsp26.7* knockdown on cold tolerance in *S. mosellana* were also investigated. The findings offer valuable molecular insights into stress tolerance associated with diapause in *S. mosellana*.

## 2. Materials and Methods

### 2.1. Insect Collection

The *S. mosellana* larvae utilized in the study were collected from natural settings, as previously described [41]. Specifically, pre-diapause larvae were obtained by dissection of *S. mosellana*-infested wheat spikes collected from wheat fields on 25 May 2022. The wheat ears containing mature third-instar larvae were bulk-collected and transferred to a field insectary in Yangling, Shaanxi, China (34°16′ N, 108°4′ E), where they were placed on damp soil to promote entry into diapause. Successful diapause entry is characterized by the presence of a larval cocoon. Prior research has shown that larvae in cocoons collected from December onwards emerge successfully as adults upon exposure to temperatures of 25 °C, indicating the termination of diapause and transition into post-diapause quiescence by December [42]. Larvae at various stages, including both larvae in diapause and those in post-diapause quiescence (i.e., cocooned larvae), as well as post-diapause developmental stages (i.e., larvae emerging from cocoons), were collected in succession by monthly sieving of the soil in the insectary between late June 2022 to early April 2023. The collected larvae were immediately frozen in liquid nitrogen and subsequently stored at −80 °C until analysis.

### 2.2. RNA Extraction, cDNA Synthesis, and gDNA Isolation

Total RNA was extracted from the entire bodies of a cohort of 20 pre-diapause *S. mosellana* larvae using TRIzol reagent (TaKaRa, Dalian, China). RNA integrity was assessed with 1% agarose gel electrophoresis, with quantification using a spectrophotometer. One microgram of total RNA was then used as a template for reverse transcription into cDNA using a PrimeScript^TM^ RT Reagent Kit with gDNA Eraser (TaKaRa, Dalian, China), as directed. For the extraction of genomic DNA, the Biospin Insect Genomic DNA Extraction Kit was utilized following the manufacturer’s instructions (Bioer Technology Co., Ltd., Hangzhou, China). 

### 2.3. Cloning of the Opening Reading Frames of SmHsp22.7 and SmHsp26.7

Based on the de novo transcriptome data of *S. mosellana* larvae obtained previously, gene-specific primers targeting *Hsp22.2* and *Hsp26.7* (Table 1) were designed to amplify the open reading frames (ORFs). The RT-PCR amplification protocol consisted of a pre-denaturation step (95 °C, 3 min), followed by 32 cycles of denaturation (95 °C, 30 s), annealing (56 °C, 30 s), and extension (72 °C, 45 s), concluding with a final extension at 72 °C for 10 min. The resultant PCR products were fractionated on 1% agarose gels, and bands of the anticipated sizes were excised and purified using a gel extraction kit (Tiangen, Beijing, China). The purified DNA fragments were thereafter integrated into a pEASYR-Blunt Zero cloning vector and transferred into Trans1-T1 competent cells.

### 2.4. Bioinformatics

The functional domains in the sequences of *S. mosellana Hsp22.2* and *Hsp26.7* were predicted using the Conserved Domain Database (https://www.ncbi.nlm.nih.gov/Structure/cdd/wrpsb.cgi, accessed on 15 October 2023), and the molecular weights and isoelectric points of the proteins were determined using the Compute pI/Mw tool (https://web.expasy.org/compute_pi/, accessed on 15 October 2023). Multiple sequence alignment was performed with DNAMAN software package (Version 8.0; Lynnon Bio-Soft, QC, Canada). Phylogenetic relationships among SmHsp22.2, SmHsp26.7, and sHsps from other insect taxa were assessed by the construction of a neighbor-joining phylogenetic tree using MEGA (version X) (https://www.megasoftware.net/, accessed on 15 May 2023). Secondary and tertiary protein structures were predicted using ESPrigt (http://espript.ibcp.fr/ESPript/cgi-bin/ESPript.cgi, accessed on 15 October 2023) and the SWISS-MODEL web server (http://swissmodel.expasy.org/, accessed on 15 October 2023), respectively. The representation and visualization of amino acid chains in the protein tertiary structures, specifically the α-helices and β-strands, were performed using PyMOL version 2.5.2 (Schrödinger, LLC, New York, NY, USA).

### 2.5. Heat/Cold Shock Treatments

*S. mosellana* typically over-summers and over-winters as cocooned larvae at depths of 3 to 10 cm beneath the soil surface [43]. The climate of Yangling district in Shaanxi Province, China (34°16′ N, 108°4′ E) is characterized by temperatures below 30 °C in summer and above 0 °C in winter. However, the soil surface temperatures can reach or exceed 45 °C in summer and may fall to −15 °C in winter, which is above the average super-cooling point (−23.6 °C) for *S. mosellana* cocoons [44]. To clarify the potential roles of *SmHsp26.7* and *SmHsp22.2* under these circumstances, cocoons were subjected to heat treatment (35‒50 °C) in August and cold treatment (−15‒0 °C) in December as outlined below.

Groups of 20 cocoons newly collected at the end of August or December were placed individually in 1.5 mL polystyrene tubes. For heat shock treatment, the tubes with the August-collected cocoons were immersed in water baths set at 35, 40, 45, or 50 °C for 1 h, or in a 35 °C water bath for 15‒90 min. Likewise, tubes containing the December-collected cocoons were exposed to incubator temperatures of 0, −5, −10, or −15 °C for 1 h or −10 °C for 15‒90 min. Untreated larvae represented the control group. After treatment, the samples were immediately frozen in liquid nitrogen, followed by storage at −80 °C until further analysis. All treatments were conducted in triplicate with 20 individuals per replicate.

### 2.6. Quantitative Real-Time PCR (qRT-PCR) Analysis

qRT-PCR analyses were conducted to assess the responsiveness of the two *S. mosellana sHsp* genes to diapause and thermal stress. qRT-PCR parameters were as follows: *Hsp22.2* (sense primer Tm = 55 °C, antisense primer Tm = 58 °C; amplicon size= 163 bp); *Hsp26.7* (sense primer Tm = 60 °C, antisense primer Tm= 60 °C; amplicon size= 195 bp); *GAPDH* (sense primer Tm = 55 °C, antisense primer Tm = 58 °C; amplicon = 86 bp). Total RNA from various samples, including larvae at distinct diapause stages and diapause larvae treated with heat or cold stress, was isolated and reverse-transcribed into cDNA as described above. The qPCR reaction volume (20 μL) contained 10 μL of 2 × SuperReal PreMix Plus (TIANGEN, Beijing, China), 1.0 μL of synthesized cDNA, 0.8 μL of each of the sense and antisense gene-specific primers (10 μM) (Table 1), and 8.2 μL of nuclease-free water. Amplification was performed on an iQ5 real-time PCR system (Bio-Rad, Hercules, CA, USA) with steps comprising pre-denaturation (95 °C, 30 s), amplification (95 °C, 5 s; 60 °C, 32 s, 35 cycles), and melting curve analysis (95 °C, 15 s; 60 °C, 60 s; 40 °C, 30 s). Each reaction was conducted in triplicate with three replicates each. RNase-free water was employed as a negative control in place of the cDNA template to ensure the accuracy and reliability of the results. The relative expression levels of *S.mosellana Hsp22.2* and *Hsp26.7* were standardized against those of the reference gene, *GAPDH* (GenBank accession number: KR733066), expressed constitutively throughout diapause, and the relative mRNA levels were quantified using the 2^−ΔΔCT^ method.

### 2.7. dsRNA Synthesis and RNA Interference

Double-stranded RNA (dsRNA)-specific primers with T7 promoter sequences for *Hsp26.7* and *Hsp22.2* (Table 1) were designed using SnapDragon dsRNA design software (https://www.flyrnai.org/snapdragon, accessed on 15 May 2023), and the corresponding gene fragments were obtained through RCR amplification as stated above. After recovery and purification of the PCR products, dsRNA synthesis was performed using the T7 High Yield Transcription Kit (Vazyme Biotech Co., Ltd., Nanjing, China), as directed, followed by further purification. A solution was prepared with 10 μL dsRNA product, 80 μL nuclease-free water, and 10 μL 3 M sodium acetate (pH 5.2). This was followed by the addition of 100 μL of phenol-chloroform, which was mixed gently and allowed to stand for 3 min before centrifugation at 12,000 rpm for 5 min. The supernatant was removed and an equal volume of isopropanol was added and incubated at −20 °C for 2 h. The material was centrifuged at 12,000 rpm for 15 min at 4 °C, and the supernatant was discarded. Next, 1 mL 75% ethanol was added, followed by centrifugation at 12,000 rpm for 15 min at 4 °C. After removal of the supernatant, the pellet was in nuclease-free water and allowed to air-dry. The synthesis and purification steps of double-strand green fluorescent protein (dsGFP) as the negative control were similar to those described above.

After quantification of the dsRNA concentration using a spectrophotometer Nanodrop2000c (Thermo Fisher Scientific, West Palm Beach, FL, USA), high-concentration dsRNAs were diluted in nuclease-free water to 10 μg/μL. Cocooned larvae that had been collected in January (post-diapause quiescent larvae) were selected for RNAi, as larvae express high levels of *SmHsp22.2* and *SmHsp26.7* at this stage (see Section 3). Based on preliminary laboratory combined analysis of post-RNAi gene-silencing efficiency and survival rates, the optimal RNAi parameters were determined to be 30 nL of 10 μg/μL double-stranded RNA (dsRNA). Thus, in this study, each cocooned larva was injected with 300 ng (30 nL) of dsRNA at the junction between the 5th and 6th abdominal segments using a Nanoject Ⅱ Auto-Nanoliter Injector (Drummond Scientific Company, Broomall, PA, USA), with corresponding amounts of dsGFP and DEPC water serving as the control groups. Following injection, the larvae were placed in Petri dishes containing moistened filter paper and incubated for 12, 24, or 48 h in an incubator at 24 ± 1 °C with 70% ± 5% relative humidity (RH) and a 16/8 h light/dark cycle. Subsequently, 20 treated individuals were selected to form one group, resulting in a total of 3 groups, to determine the gene-silencing efficiency via qPCR.

### 2.8. Cold Tolerance of Larvae After RNAi

Twenty-four hours post-RNA interference, the larvae were exposed to a temperature of −10 °C for 2 h in a low-temperature thermostat bath, as both genes exhibit peak expression levels at this temperature (see Results). The larvae were then returned to the incubator (24 ± 1 °C, 70 ± 5% relative humidity, 16:8 light:dark), and mortality was calculated every two days over a six-day period by assessing the status of the larvae. Larvae are classified as alive only if they simultaneously meet two criteria: (1) the ability to crawl after breaking the cocoon, and (2) clear responsiveness to gentle brush stimulation when motility appears ambiguous. Each group contained 40 individuals, and three separate replicates were conducted for each treatment.

### 2.9. Data Analysis

Data for different diapause stages, heat- or cold-shock treatments, and RNA interference were analyzed using one-way analysis of variance (ANOVA). Tukey’s multiple range test was applied for pairwise comparisons to assess the significance of differences between treatments with *p* < 0.05 considered statistically significant. All statistical analyses were performed using SPSS version 20.0, and the results are presented as means ± standard error (SE). For mortality scoring, all data were analyzed by Kaplan–Meier survival analysis, and differences between survival curves were assessed with the log-rank test, considering *p* < 0.05 as statistically significant. Statistical computations were performed using GraphPad Prism 9.5.0 (GraphPad Software, San Diego, CA, USA).

## 3. Results

### 3.1. Characterization of SmHsp22.2 and SmHsp26.7 cDNAs

The whole open reading frames (ORFs) of the two sHsp genes, namely *SmHsp22.2* (accession No. PQ660685) and *SmHsp26.7* (accession No. PQ660686), were obtained from transcriptome data of *S. mosellana* larvae and confirmed using RT-PCR. The ORFs of *SmHsp22.2* and *SmHsp26.7* contained 582 and 711 nucleotide pairs, respectively, corresponding to 193 and 236 amino acid residues (Figure S1). Their calculated molecular weights were 22.2 kDa and 26.7 kDa, with isoelectric points (pI) of 6.98 and 8.90, respectively. A comparison of the cDNA and gDNA sequences indicated that both genes lacked introns.

**Figure 1:**
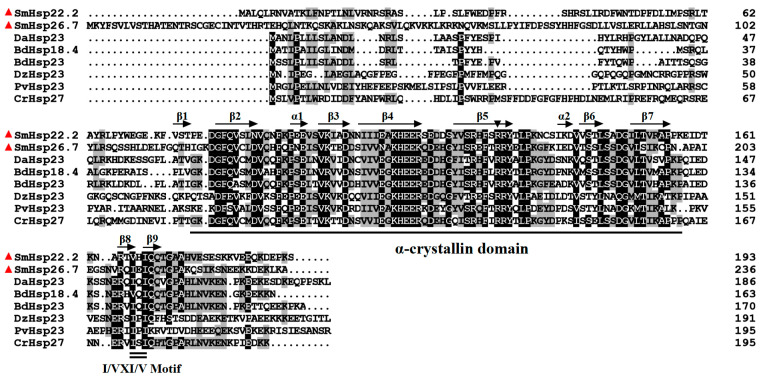
Multiple sequence alignment of SmHsp22.2 and SmHsp26.7 (red triangles) as well as sHsps from other insects. Identical and similar amino acids are distinguished by black and grey shading, respectively. The α-crystallin domain is denoted by a single underline, and the V/P/I motif is indicated by a double underline. Conserved arginine is denoted with a downward-pointing triangle. Insect species and GenBank accession numbers of sHsps shown are as follows: *Sitodiplosis mosellana* (SmHsp22.2, XMH29534; SmHsp26.7, XMH29535); *Delia antiqua* (DaHsp23, ADX36150.1); *Bactrocera dorsalis* (BdHsp18.4, ARQ14797.1; BdHsp23, XP_011198115.2); *Diamesa zernyi* (DzHsp23, UJQ69871.1); *Polypedilum vanderplanki* (PvHsp23, ADM13385.1); *Chironomus riparius* (CrHsp27, AGJ98435.1).

Analysis of domains identified the presence of the canonical α-crystallin domain in both SmHsps (a.a. 75‒155 in SmHsp22.2 and a.a. 121‒199 in SmHsp26.7). In addition, a characteristic motif I/VXI/V, situated near the C-terminus, along with an arginine residue necessary for the structural stability and chaperone function of sHsps [27,45] (Figure 1) was also conserved.

**Figure 2:**
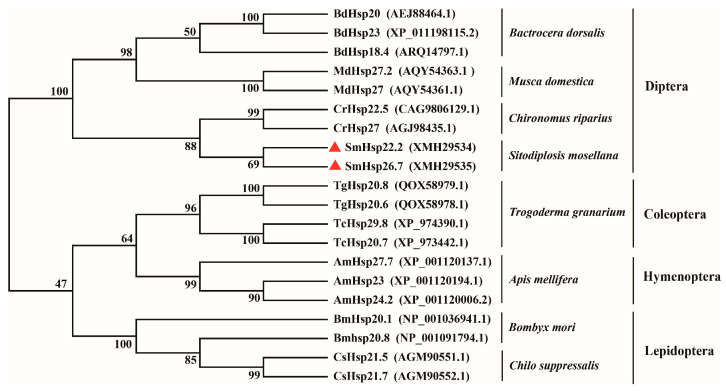
Phylogenetic neighbor-joining tree of SmHsp22.2 and SmHsp26.7 (red triangles) as well as sHsps from other insects.

BLAST (https://blast.ncbi.nlm.nih.gov/Blast.cgi, accessed on 15 May 2023) analysis indicated that SmHsp22.2 showed the highest sequence identity of 53% with Hsp23 and Hsp18.4 from *Bactrocera dorsalis*, as well as 40‒48% identity with *Chironomus riparius* Hsp27, and Hsp23 proteins from *Delia antiqua*, *Polypedilum vanderplanki*, and *Diamesa zernyi*. SmHsp26.7 displayed 57% identity with *B. dorsalis* Hsp23, and 43‒55% identity with *B. dorsalis* Hsp18.4, *Chironomus riparius* Hsp27, and Hsp23 proteins from *D. antiqua*, *P. vanderplanki*, and *D. zernyi*. The two SmHsps were 50% identical (Figure 1). The phylogenetic analysis demonstrated the clustering of sHsps from the same species, with the two SmHsps exhibiting their closest evolutionary ties to their counterparts in the Nematocera suborder of the Diptera (*C. riparius*), aligning with the canonical taxonomic relationships (Figure 2).

**Figure 3:**
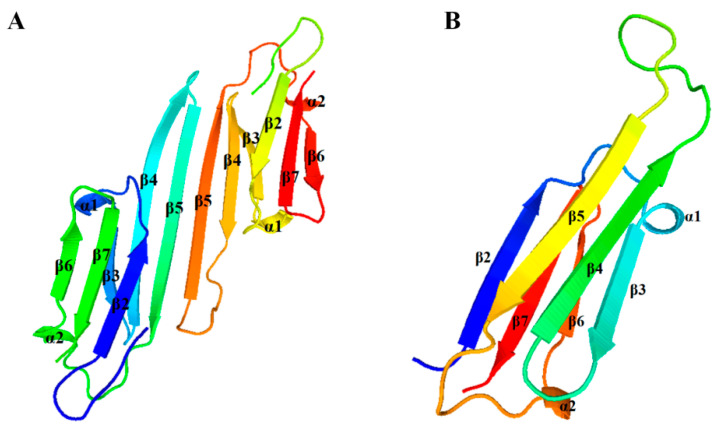
Tertiary structure prediction of SmHsp22.2 (**A**) and SmHsp26.7 (**B**). Predicted amino acid chains, specifically the α-helices and β-strands, are illustrated in a rainbow spectrum using PyMOL.

The secondary structure predictions identified six β-strands and two α-helices in the α-crystallin domains of both SmHsp22.2 and SmHsp26.7 (Figure 1). The tertiary structures of the proteins were modeled using the zebrafish (*Danio rerio*) homolog (PDB ID: 3n3e.1A) and the human (*Homo sapiens*) homolog (PDB ID: 2Wj7.1.A) as templates, due to their high sequence identity with SmHsp22.2 (45.7%) and SmHsp26.7 (56.4%), respectively. Both SmHsps contained a conserved α-crystallin domain, while SmHsp22.2 exists as a dimer and SmHsp26.7 is found as a monomer. Each monomer contained six β-strands, collectively forming a compact β-sandwich composed of two antiparallel β-sheets (Figure 3).

### 3.2. Expression of SmHsp22.2 and Hsp26.7 During Diapause

**Figure 4:**
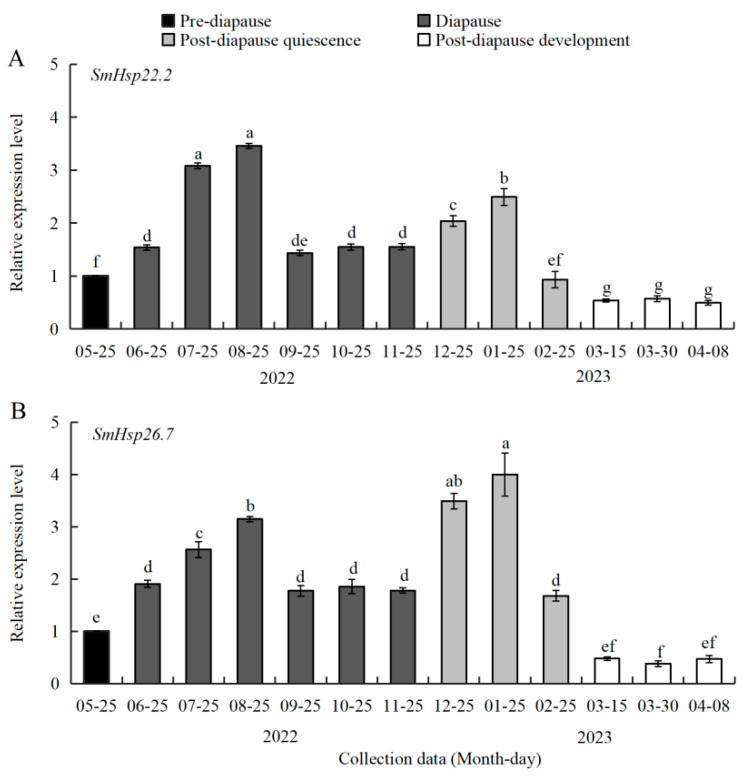
Relative transcription levels of *SmHsp22.2* (**A**) and *SmHsp26.7* (**B**) in *Sitodiplosis mosellana* larvae in pre-diapause, diapause, post-diapause quiescence, and post-diapause development (representing larvae collected during May, June‒November, December‒February, and March to early April of the following year, respectively). The transcript abundance of each tested stage was normalized to that of the pre-diapausing larvae (arbitrarily set as 1). The results are denoted as means ± SE. Bars labeled with different letters denote significant differences (Tukey’s multiple range test, *p* < 0.05).

To explore the relationship between *SmHsp26.7*/*Hsp22.2* expression and diapause, we analyzed the relative transcript levels of both genes in 3rd instar larvae of *S. mosellana* across four distinct physiological stages associated with diapause, namely, pre-diapause, diapause, post-diapause quiescence, and post-diapause development (representing larvae collected during May, June‒November, December‒February, and March to early April of the following year, respectively) (Figure 4). Expression levels of *SmHsp22.2* increased significantly after the initiation of diapause (June), persisting at high levels throughout diapause and the early-to-mid phase of post-diapause quiescence, and undergoing a sharp decline during the late post-diapause quiescence phase, ultimately reaching their lowest levels at the post-diapause developmental phase (Figure 4A).

Likewise, *SmHsp26.7* expression was low during the pre-diapause and post-diapause developmental stages, while peaking during the early-to-mid post-diapause quiescence stage (December and January), the coldest months of the year. In contrast to *SmHsp22.2*, *SmHsp26.7* expression increased markedly from July to August, surpassing the levels observed in other diapause stages (Figure 4B).

### 3.3. Expression of SmHsp22.2 and Hsp26.7 in Response to Heat Shock During Diapause

**Figure 5:**
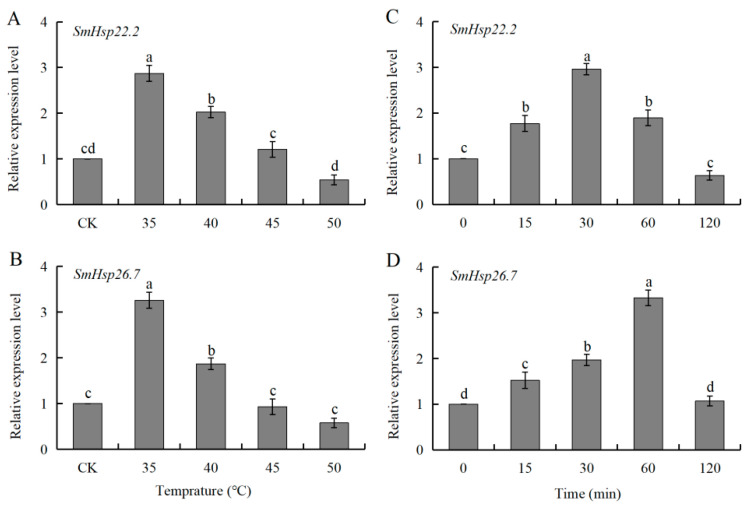
Relative transcription levels of *SmHsp22.2* and *SmHsp26.7* in response to different high temperatures (35–50 °C) for 1 h (**A**,**B**) or 35 °C for different durations (0–120 min) (**C**,**D**) in over-summering diapausing larvae of *Sitodiplosis mosellana*. The transcript abundance of each treatment was normalized to that of untreated control (CK, 0 min) (arbitrarily set as 1). The results are denoted as means ± SE. Bars labeled with different letters denote significant differences (Tukey’s multiple range test, *p* < 0.05).

The expression profiles of two *SmHsps* were markedly similar in over-summering diapause larvae after exposure to different high-temperature treatments. Compared with the control group, the expression levels of both *SmHsp22.2* and *SmHsp26.7* increased significantly in the temperature range of 35 to 40 °C, peaking at 35 °C, with increases of approximately 2.87-fold and 3.26-fold, respectively. However, no significant increase was apparent at 45–50 °C in both *S. mosellana Hsp22.2*/*26.7* genes (Figure 5A,B).

The duration of treatment also influenced the mRNA expression levels of SmHsp22.2 and SmHsp26.7. At 35 °C, the expression of both SmHsp22.2 and SmHsp26.7 rose significantly at 15 min, culminating in peaks at 30 min and 60 min, respectively (Figure 5C,D).

### 3.4. Expression of SmHsp22.2 and Hsp26.7 in Response to Cold Shock During Diapause

**Figure 6:**
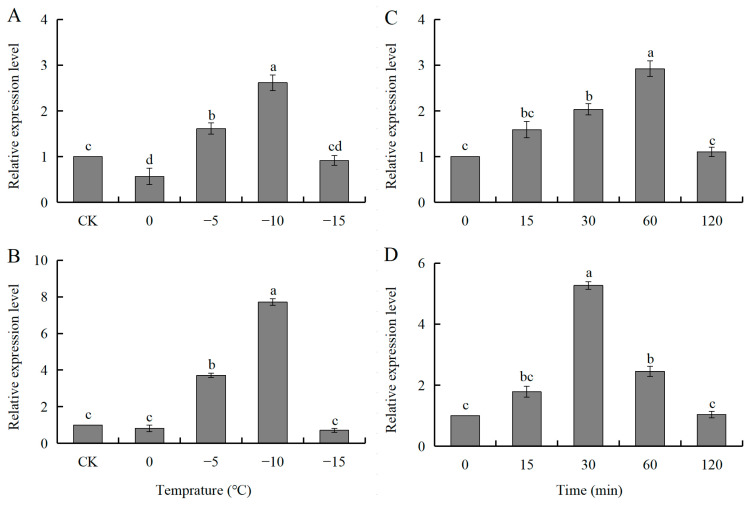
Relative transcription levels of *SmHsp22.2* and *SmHsp26.7* in response to different low temperatures (0–−15 °C) for 1 h (**A**,**B**) or −10 °C for different durations (0–120 min) (**C**,**D**) in over-wintering diapausing larvae of *Sitodiplosis mosellana*. The transcript abundance of each treatment was normalized to that of untreated control (CK, 0 min) (arbitrarily set as 1). The results are denoted as means ± SE. Bars labeled with different letters denote significant differences (Tukey’s multiple range test, *p* < 0.05).

The expression of *SmHsp22.2* and *SmHsp26.7* in over-wintering diapause larvae was also influenced by exposure to cold temperatures. At −5 °C, the expression of both *SmHsp22.2* and *SmHsp26.7* was significantly upregulated, peaking at −10 °C with approximate increases of 2.62-fold and 7.71-fold, respectively. Notably, the expression of *SmHsp22.2* was observed to decrease markedly, while that of *SmHsp26.7* showed no discernible change at 0 °C when compared to the control group (Figure 6A,B).

At −10 °C, *SmHsp22.2* expression levels rose progressively with time and were significantly upregulated at 30 min, reaching their maximum at 60 min. The expression of *SmHsp26.7*, however, showed a maximum response between 30 and 60 min of cold treatment, with expression reaching its highest point at 30 min (Figure 6C,D).

### 3.5. Effects of SmHsp22.2 and SmHsp26.7 Knockdown on Cold Tolerance in S. mosellana

**Figure 7:**
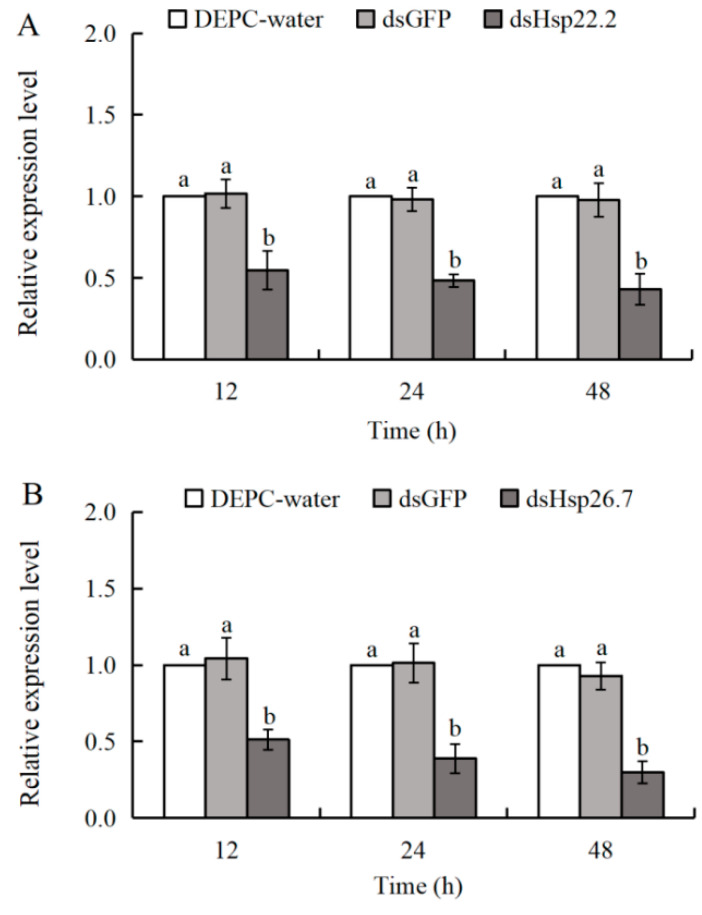
Relative expression levels of *SmHsp22.2* (**A**) and *SmHsp26.7* (**B**) at different time points after RNAi. The transcript abundance of each time point was normalized to that of the DEPC water (arbitrarily set as 1). The results are denoted as means ± SE. Bars labeled with different letters denote significant differences (Tukey’s multiple range test, *p* < 0.05).

RNAi-mediated knockdown of *SmHsp22.2*/*SmHsp26.7* led to a marked decrease in transcription levels of both genes at three time points (Figure 7). After the treatment with *dsHsp22.2*, the expression of *SmHsp22.2* dropped by 45, 52, and 57%, at 12, 24, and 48 h, respectively, compared to the DEPC-water control, as well as 47, 51, and 56% at 12, 24, and 48 h, relative to the dsGFP control (Figure 7A). Similarly, *SmHsp26.7* expression was reduced by 49, 61, and 70% compared with the DEPC-water control, and 51, 62, and 68% relative to the dsGFP control, at the same respective time points (Figure 7B). Therefore, we conclude that the knockdown of both genes was successful.

**Figure 8:**
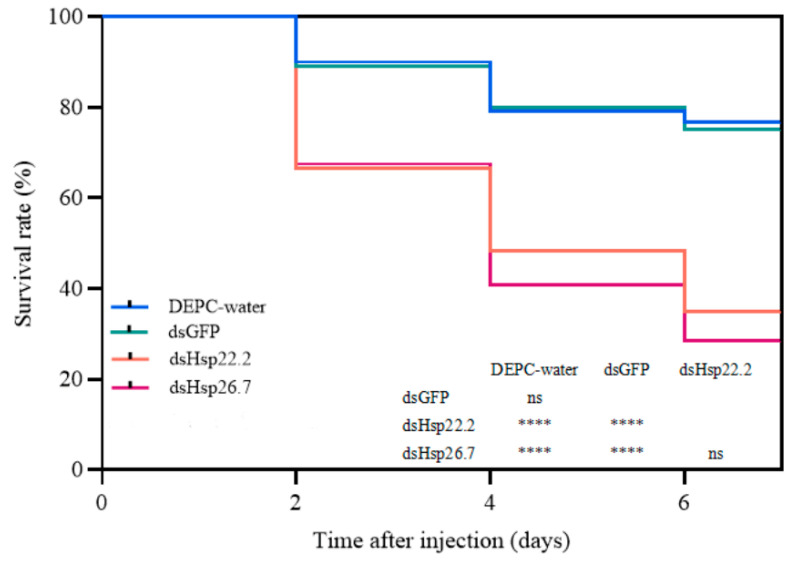
Survival curves of *Sitodiplosis mosellana* larvae injected with dsRNA. The distinct survival curves correspond to various experimental groups. DEPC-treated water and ds*GFP* functioned as the blank control and negative control, respectively. Kaplan–Meier estimators were utilized for survival curve modeling, with pairwise comparisons performed using Log-rank tests. ****, *p* < 0.0001; ns, *p* > 0.05. Each group—DEPC-water, ds*GFP*, ds*Hsp22.2*, and ds*Hsp26.7*—comprised 120 specimens, with cumulative mortality rates of 23% (28/120), 25% (30/120), 65% (78/120), and 72% (86/120) over six days, respectively.

To further investigate the roles of *SmHsp22.2* and *SmHsp26.7* in *S. mosellana* cold tolerance, larvae in which the genes had been silenced 24 h previously were placed in a low-temperature thermostat bath at −10 °C for 2 h, followed by mortality assessments every two days over a six-day period. The results showed that the survival rate of ds*Hsp22.2* or ds*Hsp26.7*-injected larvae was significantly lower than that of DEPC-water or ds*GFP* groups in 2, 4, and 6 days (*p* < 0.0001) (Figure 8), indicating the involvement of both genes in the cold resistance of *S. mosellana*.

## 4. Discussion

In addition to the two documented sHsp proteins, Hsp17.4 and Hsp20.3, in *S. mosellana* [40], this study identified two novel sHsp members from this species, namely, *SmHsp22.2* and *SmHsp26.7*. Comparative analysis of the genomic and cDNA sequences of *SmHsp22.2* and *SmHsp26.7* revealed the absence of introns in both genes. Typically, sHsps have been broadly categorized into two types, namely, orthologous types with introns and species-specific types without introns [46]. Despite the prevalence of the species-specific sHsps in insects, intron-containing genes have been observed in several species, including *Tribolium castaneum* [47], *Choristoneura fumiferana* [48], and *Mythimna separata* [49]. The evolutionary and functional diversification of sHsps in insects may thus have resulted in the emergence of genes that are either devoid of introns or possess shorter introns, thereby enhancing their capacity to respond to unfavorable environmental conditions by facilitating rapid expression [50,51].

In common with known insect sHsp proteins, SmHsp22.2 and SmHsp26.7 were found to contain all the canonical signature motifs, specifically, an α-crystalline domain comprised of several β-strands that is responsible for the molecular chaperone functionality, and the “I/VXI/V” motif that is indispensable for sustaining oligomer stability and structural assembly [23,52]. In contrast to the conserved α-crystallin domain located near the C-terminus, the N-terminal regions of sHsps exhibit substantial diversity, contributing to their ability to interact with a variety of protein partners [20]. The phylogenetic analysis demonstrated marked clustering of sHsps within the same species, which is consistent with previous findings on fungi, insects, plants, and vertebrates [53], and implies that sHsps have likely evolved through gene duplications occurring after species divergence.

Given the distinctive responsiveness and functionality of sHsp members in reacting to stress during insect diapause [3,27,28], we examined the expression patterns of two novel sHsp-encoding genes in a natural population of *S. mosellana*, rather than the commonly utilized laboratory-reared populations employed in most molecular studies on diapause. Analysis of the field-collected *S. mosellana* showed significant upregulation of both *SmHsp22.2* and *SmHsp26.7* during diapause, particularly in summer and winter (Figure 4), suggesting their potential roles in protection and cold tolerance in diapause larvae. Similar results have also been reported in other insect species, where *Hsp19.5* and *Hsp20.0* from *P. melete*, as well as *Hsp23* from *Delia antiqua*, exhibited increased expression in over-wintering and over-summering diapause pupae [27,54]. These results suggest that modulations in sHsp expression form an integral part of diapause in insects.

In natural settings, insects frequently encounter complex and severe environmental conditions, and an important adaptation strategy in insects under these conditions is modulation of the expression levels of sHsps, which exhibit varied transcriptional responses when subjected to temperature fluctuations [14,55]. The present results indicated that two *S. mosellana sHsps* were significantly upregulated in both heat-stressed over-summering larvae and cold-stressed over-wintering larvae, although this induction was transient (Figure 5 and Figure 6). It is plausible that *SmHsps* offer temporary protection for *S. mosellana* during diapause when subjected to extreme temperatures at the soil surface due to agricultural practices such as tillage [56,57]. Notably, the significant downregulation of *Hsp22.2* and *Hsp26.7* expression beyond a threshold temperature (Figure 5 and Figure 6) suggests that thermal stress causes cellular damage exceeding the protective capacity of sHsps [58], impairing their synthesis and underscoring the limited ability of insects to mitigate cellular injury through sHsp upregulation.

Overall, the transcriptional upregulation of sHsp genes in insects is closely associated with cold tolerance [14,59]. For instance, in over-wintering diapause pupae of *Sarcophaga crassipalpis*, *Hsp23* expression was found to be significantly elevated [60], and its knockdown via RNAi markedly influenced pupal survival at low temperatures [30]. Similarly, suppression of *Hsp19.0* expression in larval *Chilo suppressalis* was observed to markedly reduce their viability when exposed to subzero temperatures of −11 °C [61]. Furthermore, downregulation of *Hsp14.9*, *Hsp19.9*, *Hsp20.3*, and *Hsp24.0* in *Haemaphysalis longicornis* led to a significant decrease in their survival rates under low-temperature stress at −14 °C [13]. In this study, a temperature of −10 °C was selected to analyze the functions of *S. mosellana Hsp22.2* and *Hsp26.7*, as the peak expression levels of both genes occur at this temperature (Figure 6). After injection of *dsHsp22.2*/*26.7*, the larvae exhibited significantly lower survival rates compared to the DEPC-water and dsGFP control groups (Figure 8), suggesting that both sHsp genes are essential for protecting *S. mosellana* from the adverse effects of low temperature. These findings highlight potential molecular targets for RNA interference (RNAi)-based pest management strategies. Nonetheless, practical application necessitates further refinement of delivery vectors and comprehensive ecological risk assessments prior to field deployment. Thus, there is still a long way to go before *Sitodiplosis mosellana* can be effectively controlled.

## 5. Conclusions

The diapause-induced upregulation of *SmHsp22.2* and *SmHsp26.7*, their induction upon sublethal heat (35–40 °C) and moderate cold (−10 °C), and gene-specific RNAi-induced cold sensitivity are strong results that enhance knowledge about sHsp function in insect stress physiology. These findings have potential practical relevance to predicting the dynamics of pest populations under climate variability and indicate that *SmHsps* could be targeted with RNAi-based biocontrol in wheat systems; however, field validation is necessary.

## Figures and Tables

**Table 1:** Primer sequences used in this study.

Primer Name	Sequence (5′ to 3′)	Purpose
Hsp22.2 sense	CTAAAGTGAAGTAGAAAAAATGG	ORF and gDNA cloning
Hsp22.2 antisense	GCATCACATCTTTTACATTCC	
Hsp26.7 sense	ATGAAGTATTTCTCCGTTTTGG	
Hsp26.7 antisense	TTAGGCCTTTAGTTTTTCATCC	
dsHsp22.2 sense	taatacgactcactatagggTGTTTCACGACACTTCAGCC	dsRNA synthesis
dsHsp22.2 antisense	taatacgactcactatagggGGCTCCAGTTTGTTGGATGT	
dsHsp26.7 sense	taatacgactcactatagggAGATTGTTGGCTCACTCGCT	
dsHsp26.7 antisense	taatacgactcactatagggCTTGGCGTTCACCACAATCG	
dsGFP sense	taatacgactcactatagggTGACCACCCTGACCTAC	
dsGFP antisense	taatacgactcactatagggTTGATGCCGTTCTTCTGC	
Hsp22.2 sense	ATTGCCATCGTTGTTCTG	qPCR
Hsp22.2 antisense	TCCATCTTCGGGTGTGCT	
Hsp26.7 sense	CGATTGTGGTGAACGCCAAG	
Hsp26.7 antisense	CAATTTGGCGCACGTTGGAT	
GAPDH sense	CCATCAAAGCAAGCAAGA	
GAPDH antisense	CAGCACGGAGCACAAGAC	

## Data Availability

The original contributions presented in this study are included in the article/Supplementary Materials. Further inquiries can be directed to the corresponding author.

## Supplementary Materials

The following supporting information can be downloaded at https://www.mdpi.com/article/10.3390/insects16070649/s1, Figure S1. Nucleic acid and deduced amino acid sequences of *SmHsp22.2* and *SmHsp26.7* in *Sitodiplosis mosellana*. Initiation codons (ATG) and termination codons (TAA) were indicated by boxes. The α-crystallin domain was highlighted by shading. I/VXI/V motif was indicated with a single underline.
