# Supplementary material for: Characterization and Functional Analysis of Small Heat Shock Protein Genes (Hsp22.2 and Hsp26.7) in Sitodiplosis mosellana Diapause
Source: Insects. 2025 Jun 20;16(7):649. doi: 10.3390/insects16070649 (PMC12296141; doi:10.3390/insects16070649)
Supplement: Supplementary file 1 [file insects-16-00649-s001.zip › insects-3699650-supplementary.pdf]

*insects*

MDPI

```
SmHsp22.2

ATGGCTTTACAATTGAGAAACGTTGCCACCAAATTGTTTAATCCAACATTGAATTTGGTGCGAAACCGTTCACGAGCATCATTGCCATCGTTGTTCTGGGAAGATCCATTCAGAAGTCAT   120
 M  A  L  Q  L  R  N  V  A  T  K  L  F  N  P  T  L  N  L  V  R  N  R  S  R  A  S  L  P  S  L  F  W  E  D  P  F  R  S  H    40
CGTAGTTTGATTAGAGATTTTTGGAATACGGATCCATTTGACTTGATAATGCCAAGTCGACTAACTGCATACCGGCTGCCTTATTGGGAAGGTGAAAAATTCGTGAGCACACCCGAAGAT   240
 R  S  L  I  R  D  F  W  N  T  D  P  F  D  L  I  M  P  S  R  L  T  A  Y  R  L  P  Y  W  E  G  E  K  F  V  S  T  P  E  D    80
GGATTTCAAGTGTCGTTGAATGTGCAAAACTTTAAACCAGAAGAAGTTTCTGTGAAAATCGCCGATAATAACATCATCATTGAGGCGAAACATGAAGAACGTAGTGAAGACGATAGCTAT   360
 G  F  Q  V  S  L  N  V  Q  N  F  K  P  E  E  V  S  V  K  I  A  D  N  N  I  I  I  E  A  K  H  E  E  R  S  E  D  D  S  Y   120
GTTTCACGACACTTCAGCCGACGATACACATTGCCCAAGAATTGCAGCATCAAAGATGTGGTCTCTACCTTGTCGGCTGATGGCATTCTAACCGTTCGAGCTCCACCAAAGGAAATTGAC   480
 V  S  R  H  F  S  R  R  Y  T  L  P  K  N  C  S  I  K  D  V  V  S  T  L  S  A  D  G  I  L  T  V  R  A  P  P  K  E  I  D   160
ACCAAAAATGCACGAACAGTTCACATCCAACAAACTGGAGCCGCTCATGTCGAATCAGAGTCAAAAAAAGTTGAGGAACAAAAAGACGAACCAAAAAGTTAA                     600
 T  K  N  A  R  T  V  H  I  Q  Q  T  G  A  A  H  V  E  S  E  S  K  K  V  E  E  Q  K  D  E  P  K  S  *                     200

SmHsp26.7

ATGAAGTATTTCTCCGTTTTGGTGTCGACTCATGCAACCGAAAATACGCGATCCTGTGGCGAATGTATAAATACCGTGACGCATCGCACTGAGCATCAGTTGAATACAAAGCAAAGCAAA   120
 M  K  Y  F  S  V  L  V  S  T  H  A  T  E  N  T  R  S  C  G  E  C  I  N  T  V  T  H  R  T  E  H  Q  L  N  T  K  Q  S  K    40
GCAAAGTTAAACTCAAAACAAGCGAAAAGTGTTCTTCAAAAAGTGAAAAAGTTGAAAAGAAAAAATCAAGTAAAAATGTCACTTTTGCCATACATTTTCGACCCATCCAGCTACCATCAT   240
 A  K  L  N  S  K  Q  A  K  S  V  L  Q  K  V  K  K  L  K  R  K  N  Q  V  K  M  S  L  L  P  Y  I  F  D  P  S  S  Y  H  H    80
TTTGGTTCAGATTTGCTGGTTTCTCTTGAAAGATTGTTGGCTCACTCGCTATCAAACACCGGCAATTATTTGCGATCGCAATCGTCGCATTTGGACGAGCTATTTGGACAGACGCATATC   360
 F  G  S  D  L  L  V  S  L  E  R  L  L  A  H  S  L  S  N  T  G  N  Y  L  R  S  Q  S  S  H  L  D  E  L  F  G  Q  T  H  I   120
GGAAAGGACGGTTTCCAAGTCTGTCTCGACGTTCAACACTTCCAACCGAACGAGATCTCGGTGAAAACGGAAGACGATTCGATTGTGGTGAACGCCAAGCACGAAGAGAAACAAGACGAA   480
 G  K  D  G  F  Q  V  C  L  D  V  Q  H  F  Q  P  N  E  I  S  V  K  T  E  D  D  S  I  V  V  N  A  K  H  E  E  K  Q  D  E   160
CACGGTTACATTTCGCGAGAATTTACTCGTCGCTATGAACTACCAAAAGGATTCAAGATTGAAGACGTAACATCAAGTCTCTCATCTGATGGCGTTCTCTCGATTAAATGCCCTAACGCT   600
 H  G  Y  I  S  R  E  F  T  R  R  Y  E  L  P  K  G  F  K  I  E  D  V  T  S  S  L  S  S  D  G  V  L  S  I  K  C  P  N  A   200
CCGGCCATCGAAGGATCCAACGTGCGCCAAATTGAAATCCAACAAACTGGCCCAGCCAAGCAAAGCATTAAGAGCAACGAAGAAAAGAAGGATGAAAAACTAAAGGCCTAA            720
 P  A  I  E  G  S  N  V  R  Q  I  E  I  Q  Q  T  G  P  A  K  Q  S  I  K  S  N  E  E  K  K  D  E  K  L  K  A  *            240
```

**Figure S1.** Nucleic acid and deduced amino acid sequences of *SmHsp22.2* and *SmHsp26.7* in *Sitodiplosis mosellana*. Initiation codons (ATG) and termination codons (TAA) were indicated by boxes. The $\alpha$-crystallin domain was highlighted by shading. I/VXI/V motif was indicated with a single underline.

 https://doi.org/10.3390/insects16070649
